# Design and Immunological Evaluation of a Hybrid Peptide as a Potent TLR2 Agonist by Structure-Based Virtual Screening

**DOI:** 10.3389/fcell.2021.620370

**Published:** 2021-02-11

**Authors:** Lulu Zhang, Xubiao Wei, Rijun Zhang, Paul E. Mozdziak, Dayong Si, Baseer Ahmad, Qiang Cheng, Yucui Tong

**Affiliations:** ^1^State Key Laboratory of Animal Nutrition, College of Animal Science and Technology, China Agricultural University, Beijing, China; ^2^School of Pharmaceutical Sciences, Tsinghua University, Beijing, China; ^3^Prestage Department of Poultry Science, College of Agriculture and Life Sciences, North Carolina State University, Raleigh, NC, United States

**Keywords:** immunoregulatory activity, TLR2, molecule docking, cyclophosphamide, NF-κB signaling

## Abstract

Immunity is a versatile defensive response that is involved in protecting against disease by identifying and destroying self and non-self harmful substances. As a state of temporary or permanent immune dysfunction, immunosuppression can make an organism more susceptible to infection, organ injury, and cancer due to damage to the immune system. It has taken a long time to develop new immunomodulatory agents to prevent and treat immunosuppressive diseases. In recent years, Toll-like receptor 2 (TLR2) agonists have been reported to have profound effects on the immune system, and they are regarded as potent immunomodulatory candidates. TP5 and LL-37, the potent immunomodulatory agents, have been reported to produce a robust innate immune response by binding to TLR2. However, their development has been weakened by several concerns, such as potential cytotoxicity, weak physiological stability and poor immunomodulatory activity. To overcome these challenges, hybridization has been proposed. Therefore, six hybrid peptides (LTPa, LTPb, LTPc, TPLa, TPLb, and TPLc) were designed by combining the full-length TP5 with a characteristic fragment of LL-37 that included LL-37 (13–36), LL-37 (17–29), and LL-37 (13–31). LTPa, the most potent TLR2 agonist, was simply and effectively screened by molecular docking and *in vitro* experiments. Furthermore, the immunomodulatory effects of LTPa were confirmed by a CTX-immunosuppressed murine model, which demonstrated that LTPa successfully inhibit immunosuppression, increased immune organ indices, enhanced DC maturation, regulated T lymphocyte subsets, and increased cytokine and Ig contents. Our study also revealed that the immunomodulatory effects of LTPa are associated with binding to TLR2, forming TLR2 clusters, and activating the NF-κB signaling pathway.

## Introduction

Immunity is a versatile defensive response that is involved in protecting against disease by identifying and destroying self and non-self harmful substances or microbiological toxins (Hooper et al., 2012). As a state of temporary or permanent immune dysfunction, immunosuppression can make an organism more susceptible to infection, organ injury, and cancer due to damage to the immune system (Goldszmid et al., 2014). Therefore, it is necessary to develop a new immunomodulatory agent to prevent and treat immunosuppressive diseases.

Toll-like receptors (TLRs) are thought of as pathogen recognition receptors for exogenous pathogens and have also been shown to play prominent roles in host defense and in regulating immune responses (Akira et al., 2006). TLR2 is an important signal transduction molecule that is expressed on dendritic cells (DCs), macrophages and activated CD4 + T cells (Komai-Koma et al., 2004). TLR2 activates the secretion of proinflammatory cytokines such as tumor necrosis factor alpha (TNF-α), interleukin-6 (IL-6), and IL-1β from monocytes, macrophages and dendritic cells, thereby triggering the activation of the immune response (Oliveira-Nascimento et al., 2012). Therefore, TLR2 agonists, such as β-glucan (Quayle et al., 2015) and Pam_3_CSK_4_ (Su et al., 2019), are promising candidates as vaccine adjuvants and pharmaceuticals that support immunotherapies.

Thymopentin (TP5), the Arg32–Tyr36 fragment derived from thymopoietin, was able to restore the thymic atrophy induced by immunosuppressants (Goldstein et al., 1979; Singh et al., 1998). TP5 was found to exert its immunoregulatory activity by binding TLR2 (Li et al., 2013). Additionally, TP5 plays an important role in T-lymphocyte maturation and differentiation, thereby regulating immunity (Mendling and Koldovsky, 1996; London et al., 2011). Overall, TP5 is used in the treatment of immunodeficiencies, malignancies, and infections due to its immunoregulatory activity and low cytotoxicity.

LL-37, a human cationic host-defense peptide, has been reported to have a robust innate immune response by binding to TLR2 (Mansour et al., 2014; Zhang et al., 2016; Agier et al., 2018). In addition, LL-37 stimulates the production of various immunoregulatory mediators, proinflammatory cytokines, and chemokines (Scott et al., 2002; Tjabringa et al., 2003; Bowdish et al., 2004). Therefore, LL-37 could prevent or attenuate immunosuppression.

TP5 plays a vitally important role in the process of immune enhancement. However, the development of TP5 has been weakened by its short half-life, which decreases its efficacy and bioavailability (Gonser et al., 2004; Hu et al., 2016). LL-37 has a long half-life, but its clinical development has been hampered by its cytotoxicity (Anders et al., 2018). As a simple and effective strategy that could combine the advantages of different native peptides (Li et al., 2014; Wei et al., 2018), hybridization has been proposed to obtain a peptide with more potent immunoregulatory activity but minimal cytotoxicity. As previously reported, LL-37 (13–36) (Mookherjee et al., 2006; Molhoek et al., 2009), LL-37 (17–29) (Li et al., 2006; Tan et al., 2017), and LL-37 (13–31) (Wei et al., 2018) exhibit robust immunoregulatory activity. Therefore, six hybrid peptides were designed by combining the full-length TP5 (Gonser et al., 2004; Hu et al., 2016) with a characteristic fragment of LL-37 that included LL-37 (13–36) (Mookherjee et al., 2006; Molhoek et al., 2009), LL-37 (17–29) (Li et al., 2006; Tan et al., 2017), and LL-37 (13–31) (Wei et al., 2018). The hybrid peptides were screened by molecular docking and *in vitro* experiments based on their immunoregulatory activity and cytotoxicity. The immunoregulatory mechanisms of the best hybrid peptide were further analyzed by exploring the molecular basis of its immunoregulatory effects using a cyclophosphamide (CTX)-immunosuppressed murine model. The objective of the study was to develop a TLR agonist using a novel screeing strategy. The aim was to develop a model system that could lead to a potential therapeutic to improve the immune system in immunocompromised individuals.

## Materials and Methods

### Hybrid Peptide Design

The hybrid peptides LL-37-TP5a (LTPa, IGKEFKRIVQRIK DFLRNLVPRTERKDVY), LL-37-TP5b (LTPb, FKRIVQRIKD FLRRKDVY), LL-37-TP5c (LTPc, IGKEFKRIVQRIKDFL RNLRKDVY), TP5-LL-37a (TPLa, RKDVYIGKEFKRIVQRIKD FLRNLVPRTE), TP5-LL-37b (TPLb, RKDVYFKRIVQRIK DFLR), and TP5-LL-37c (TPLc, RKDVYIGKEFKRIVQRIKDFL RNL) were designed by combining TP5 (RKDVY) with three active centers of LL-37, including LL-37a (IGKEFKRIVQRIKDFLRNLVPRTE), LL-37b (FKRIVQRI KDFLR), and LL-37c (IGKEFKRIV QRIKDFLRNL). The key physicochemical parameters of the peptides were calculated online using ExPASy Proteomics Server^1^. The initial 3D structures of all these hybrid peptides were generated through MOE suit and I-TASSER online server I-TASSER^2^.

### Circular Dichroism Spectra

The Circular dichroism (CD) spectroscopy of the hybrid peptides was performed at 25°C using a Jasco-810 spectropolarimeter (Jasco, Tokyo, Japan). The peptides were dissolved in 10 mM PBS (pH 7.4, mimicking an aqueous environment) and 50% (v/v) TFE (mimicking the hydrophobic environment of the cell membrane) at a concentration of 0.1 mg/mL. The measurements were recorded at wavelengths ranging from 190 to 250 nm.

### Molecular Docking

The initial structures of the hybrid peptides were then subjected to molecular docking. The published TLR2 structure (PDB code: 1FYW) was used as a receptor structure for molecule docking. All the default optimization parameters were used for the iterated search in Vina. ZDOCK3.0.2 was used to produce the initial TLR2-hybrid peptide complex. A total of 3,600 decoy structures of each TLR2-hybrid peptide complex were predicted through the rigid-binding option in ZDOCK. The decoy with the lowest energy was selected for the flexible docking study. One hundred docking runs of each molecule were performed by flexpepdock^3^. The most plausible docking conformation with the lowest interface energy was chosen for hybrid peptide scanning.

### Peptide Synthesis

All of the peptides used in this study were synthesized and purified by KangLong Biochemistry (Jiangsu, China). The purity of the peptide was determined to be greater than 95% by high-performance liquid chromatography (HPLC). The peptides were identified through mass spectrometry (MS) and dissolved in endotoxin-free water (Supplementary Figure S1).

### Cell Culture

The murine macrophage cell line RAW264.7 (Shanghai Cell Bank, Shanghai, China) was cultured in Dulbecco’s modified Eagle’s medium (DMEM; HyClone Logan, UT, United States) and incubated in a humidified atmosphere of 5% CO_2_ at 37°C. DMEM was supplemented with 10% (v/v) fetal bovine serum (FBS; Gibco, Foster, CA, United States) and 1% (v/v) penicillin/streptomycin (HyClone).

### Cell Viability Assay

The cytotoxicity of the peptides against RAW264.7 cells was measured using a Cell Counting Kit-8 (CCK-8) Assay Kit (Dojindo) as previously described (Liu Z.H. et al., 2013). Briefly, cells were seeded in 96-well plates at a final density of 2.0 × 10^4^ cells/well overnight. The cells were incubated with different concentrations of each peptide (0–80 μg/mL) for 24 h and then further incubated with CCK-8 solution for 4 h at 37°C in darkness. The OD value was then measured using a microplate reader (TECAN, Switzer) at 450 nm. Cell viability was calculated using the equation:

$$Cellviability\left(\%\right)=\frac{\text{OD}\,450\,\left(\text{sample}\right)}{\text{OD}\,450\,\left(\text{control}\right)}\times 100\%$$

Where OD450 (sample) is the OD value at 450 nm by peptide-treated cells and OD450 (control) is the OD value at 450 nm by the cells without peptide treatment.

### *In vitro* Immunomodulatory Activity of Peptides

RAW264.7 cells were seeded at a density of 1.0 × 10^6^ cell/mL and treated with (10 μg/mL) or without peptides in each well of a 6-well plate for 12 h at 37°C in a humidified incubator with 5% CO_2_. After treatment, TNF-α, IL-6 and IL-1β secretion in the cell supernatants was assessed using ELISA kits according to the manufacturer’s instructions.

### Experimental Animals

BALB/c mice (female, 6–8 weeks; Charles River, Beijing, China) used in this study were maintained in a specific-pathogen-free facility (55 ± 10% humidity and 22 ± 1°C) during the experiments. These experiments were conducted with approval from the Institutional Animal Care and Use Committee of China Agricultural University (certificate of the Beijing Laboratory Animal employee, ID: 18086).

After a 7-day adaptation period, the mice were randomly divided into five groups (*n* = 12 in each group): (1) control group: physiological saline was injected intraperitoneally once daily; (2) cyclophosphamide (CTX) group: CTX (80 mg/kg mouse weight; Sigma-Aldrich, St. Louis, MO, United States) was injected intraperitoneally once daily for the first 3 days. From days 4 to 10 (7 days), physiological saline was injected intraperitoneally into mice once daily; and (3) CTX + peptide (LL-37, TP5, or LTPa) groups: CTX (80 mg/kg mouse weight) was injected intraperitoneally once daily for three consecutive days. From days 4 to 10, peptide (10 mg/kg mouse weight) was injected intraperitoneally into mice once daily. Twenty-four hours after the last drug administration, all the mice were weighed and then euthanized, and their tissues and blood were collected for further study. The body weight index was calculated according to the formula:

$$\mathrm{Index}\left(\%\right)=\frac{\mathrm{final}\,\mathrm{body}\,\mathrm{weight}-\mathrm{inital}\,\mathrm{body}\,\mathrm{weight}}{\mathrm{initial}\,\mathrm{body}\,\mathrm{weight}}\times 100$$

The thymus and spleen indices were measured by the following formula:

$$\mathrm{Index}\,\left(\mathrm{mg}/g\right)=\frac{\mathrm{weight}\,\mathrm{of}\,\mathrm{thymus}\,\mathrm{or}\,\mathrm{spleen}}{\mathrm{body}\,\mathrm{weight}}$$

### Peripheral Blood T Lymphocyte Subpopulations Analyzed by Flow Cytometry

Erythrocytes were aseptically obtained from the different groups of mouse peripheral blood and lysed with RBC lysis buffer. The remaining cells were washed with PBS and resuspended in PBS. The cells were combined with peridinin-chlorophyll-protein complex (PerCP)-conjugated anti-mouse CD3 +, allophycocyanin (APC)-conjugated anti-mouse CD4 +, and fluorescein isothiocyanate (FITC)-conjugated anti-mouse CD8 + (BD Pharmingen, United States) for 30 min at 4°C. The T lymphocyte subpopulations were analyzed by flow cytometry.

### Phenotypic Analysis by Flow Cytometry

Mouse peripheral blood mononuclear cells (PBMCs) were obtained from the different groups of mice through Ficoll density gradient centrifugation. The cells were resuspended in PBS with 5% FBS and incubated with 10% (v/v) normal goat serum for 15 min at 4°C. The cells were combined with FITC-conjugated antibodies specific for MHC class II (MHC-II; BD Pharmingen, United States). The surface expression of MHC-II was determined by flow cytometry.

### Serum Cytokine and Immunoglobulin (Ig) Measurements by ELISA

Serum was collected from blood and then assayed by ELISA kits according to the protocol supplied by the manufacturer (eBioscience, San Diego, United States) to determine the secretion of IgG, IgA, IgM, TNF-α, IL-6, and IL-1β.

### Blocking Experiments

For TLR2 blocking experiments, RAW264.7 cells were cultured in 24-well plates overnight at 37°C. The mouse IgG2a isotype control antibody or anti-mouse TLR2 (C9A12; InvivoGen, San Diego, CA, United States) was added to the cell cultures 1 h before being treated with LTPa for 24 h at 37°C. Then, the levels of TNF-α, IL-6, and IL-1β in the culture supernatant were analyzed.

### Molecular Dynamics Simulation

To assess the binding affinity of LTPa with TLR2, the docking pose of LTPa-TLR2 was assessed by RosettaDock (version 3.5), and the missing hydrogen atoms in this system were added by Maestro at pH 7.0 (Sastry et al., 2013). The pose with the lowest binding energy was selected for further study.

The best binding poses of LTP with TLR2 were subjected to molecular dynamics (MD) simulation under AMBER14 (Duan et al., 2003; Wang et al., 2005). The GAFF and FF14SB force fields were used for peptides and proteins, respectively. The LTP-TLR2 complex was solvated in a cubic box of TIP3P water with a minimum distance of 10 Å and then neutralized by adding an appropriate number of Na^+^ and Cl^–^ atoms (Jorgensen et al., 1983). The first 1,000 steps were used to minimize the system by the conjugate gradient algorithm. The system was then heated gradually at a constant number, pressure, and temperature (NPT) for 100 ps (Parrinello and Rahman, 1981). After that, a 60 ns MD simulation was performed under a constant number, volume, and temperature (NVT) ensemble. The molecular mechanics Poisson-Boltzmann accessible surface area (MM-PBSA) binding free energy was calculated for the LTP-TLR2 complex (Massova and Kollman, 2000). The particle-mesh Ewald method was employed to address the long-range electrostatic interactions.

### Surface Plasmon Resonance

Surface plasmon resonance (SPR) experiments were carried out using a Biacore X100 instrument (GE Healthcare, Pittsburgh, PA, United States) by standard amine coupling. TLR2 (R&D Systems, Minneapolis, MN, United States) was amine-coupled to a Biacore CM5 sensor chip (Bio-Rad). The peptide was diluted to varying concentrations (0, 1.25, 2.5, 5, and 10 mM) in running buffer (PBS containing 0.05% Tween 20) and run sequentially over immobilized TLR2. Binding curves were processed for the initial injection alignment and baseline, and a reference-subtracted sensorgram was globally fitted to the curves to describe a homogeneous 1:1 model. Data analysis was conducted using ProteOn manager software (version 2.0). Data from the protein surfaces were grouped together to fit the association kinetic rate constant (*K*a) and the dissociation rate constant (*K*d). The equilibrium dissociation constant (*K*D) for the peptide-TLR2 interaction was calculated as follows:

$$\mathrm{KD}=\frac{\mathrm{Kd}}{\mathrm{Ka}}$$

### Confocal Laser-Scanning Microscopy

RAW264.7 cells were treated with or without 10 μg/mL LTPa for 1 h at 37°C. The cells were then incubated (30 min, on ice) with anti-mouse monoclonal antibody TLR2 or isotype control (IgG) (eBioscience) before washing with PBS. Then, the cells were stained with a FITC–conjugated anti-mouse IgG antibody for 30 min on ice (Jackson Laboratories). After washing with PBS and fixing with paraformaldehyde, the cell nuclei were stained with DAPI (1:500 dilution in PBS) (Sigma-Aldrich). The cells were then imaged with the Leica TCS SP5 confocal system (Leica Microsystems Ltd.). LAS AF software was used to process the images and quantify the fluorescence. In addition, to generate a fluorescence histogram profile, a line was drawn along the cell surface. Fluorescence intensities higher than 40 arbitrary units (isotype control staining) were considered clusters of TLR2 molecules.

### Western Blot Analysis

Whole proteins were extracted from the serum using a whole-protein extraction kit (Nanjing KeyGEN Biotech, Nanjing, China). The protein concentrations were determined with the BCA Assay (KeyGEN Biotech, Nanjing, China) according to the manufacturer’s instructions. Equivalent amounts of protein were separated by SDS-PAGE and blotted onto polyvinylidene fluoride membranes (Bio-Rad). After blocking with 5% non-fat dried milk in TBS/0.05% Tween-20 for 2 h at room temperature, the membranes were immunoblotted with primary specific antibodies (anti-TLR2, anti-TRAF6, anti-TAK1, anti-IκB-α, anti-p-IκB-α, anti-NF-κB (p65), anti-p-NF-κB (p-p65), and anti-β-actin (Abcam, United Kingdom) at 4°C overnight. After washing with TBST, the membranes were incubated with horseradish peroxidase (HRP)-conjugated secondary antibody (HuaAn, Hangzhou, China) for 1 h at room temperature. Proteins were visualized using a ChemiDoc^TM^ MP Imaging System (Bio-Rad). Quantitative data were evaluated by means of ImageJ software.

### Statistics

All experiments were carried out with at least three biological replicates. The data are presented as the means ± SD. Statistical comparisons were performed by Student’s *t* test and one-way ANOVA using GraphPad Prism 7 software.

## Results

### Screening of Immunomodulatory Peptides

**FIGURE 1:**
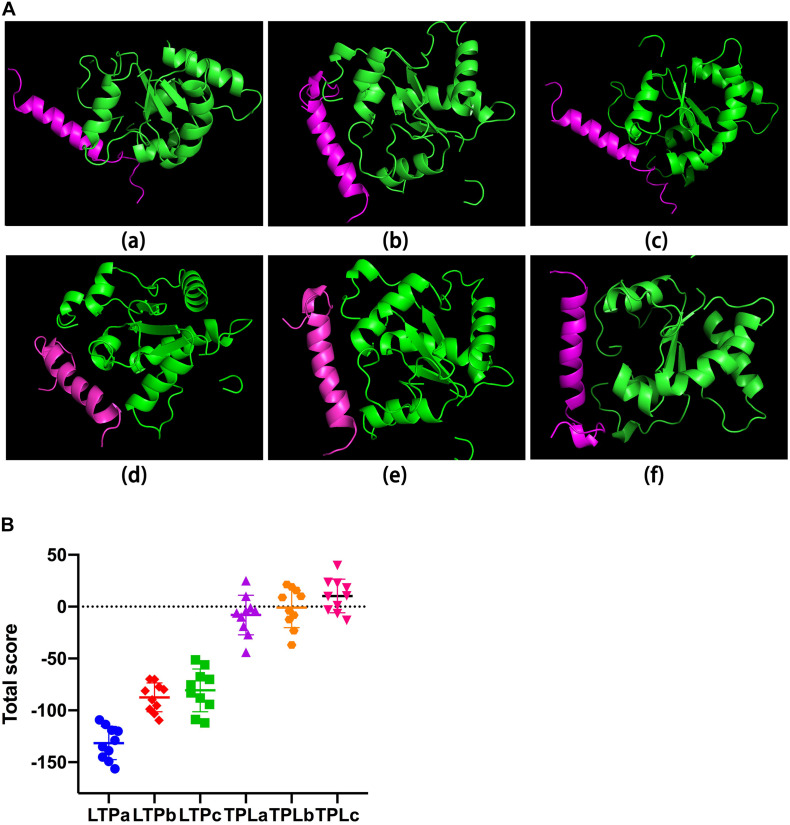
The binding of ability of the hybrid peptides to TLR2. **(A)** The overall structure of the peptide-TLR2 complex. The green ribbons represent TLR2, and the red ribbons represent the hybrid peptide. **(B)** Energy plot. Ten out of 100 decoy structures from the hybrid peptide-TLR2 docking study by flexpepdock. Data are means ± SD.

Table 1 shows the sequences of the hybrid peptides and their parental peptides. To scan a hybrid peptide with desired immunomodulatory activity, the binding ability of the hybrid peptides to TLR2 was determined by molecular docking. LTPa had lower binding energy than the other hybrid peptides (Figure 1), and its total score was lower than -100.

**TABLE 1:** The sequence of the peptides.

Peptides	Sequence
LL-37	LLGDFFRKSKEKIGKEFKRIVQRIKDFLRNLVPRTES
TP5	RKDVT
LTPa	IGKEFKRIVQRIKDFLRNLVPRTERKDVT
LTPb	FKRIVQRIKDFLRRKDVT
LTPc	IGKEFKRIVQRIKDFLRNLRKDVT
TPLa	RKDVTIGKEFKRIVQRIKDFLRNLVPRTE
TPLb	RKDVT FKRIVQRIKDFLR
TPLc	RKDVTIGKEFKRIVQRIKDFLRNL

**FIGURE 2:**
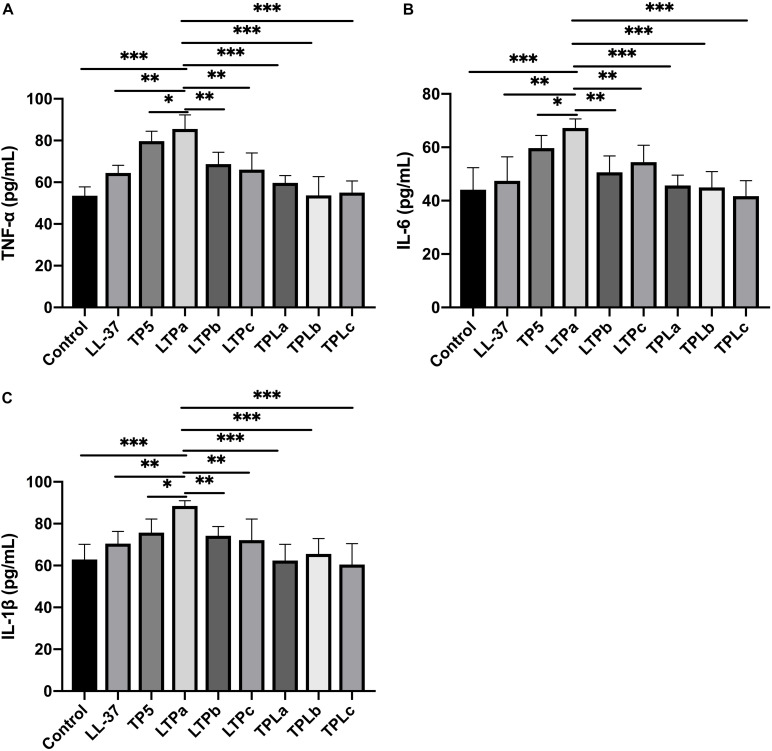
The *in vitro* immunomodulatory activity of the hybrid peptides. The mouse macrophages (RAW264.7) were treated with 10 μg/mL hybrid peptides for 12 h. After treatment, TNF-α **(A)**, IL-6 **(B)** and IL-1β **(C)** secretion in the cell supernatants was assessed using ELISA kits. The mean values ± SD from three independent experiments were used to express the data. ^∗^*p* ≤ 0.05, ^∗∗^*p* ≤ 0.01, and ^∗∗∗^*p* ≤ 0.001.

The immunomodulatory activities of these hybrid peptides were then evaluated by *in vitro* experiments, which suggested that the hybrid peptides LTPa, LTPb and LTPc and their parental peptides caused remarkable increases in TNF-α (Figure 2A), IL-6 (Figure 2B), and IL-1β (Figure 2C) production. However, TPLa, TPLb, and TPLc exhibited no significant increases in these cytokines. In addition, LTPa significantly increased the secretion of TNF-α, IL-6, and IL-1β in comparison with the other peptides tested. Collectively, these data suggest that LTPa, the most active peptide, was worthwhile focus for further immunomodulatory experiments.

### Cytotoxicity on RAW264.7 Macrophage Cells

**FIGURE 3:**
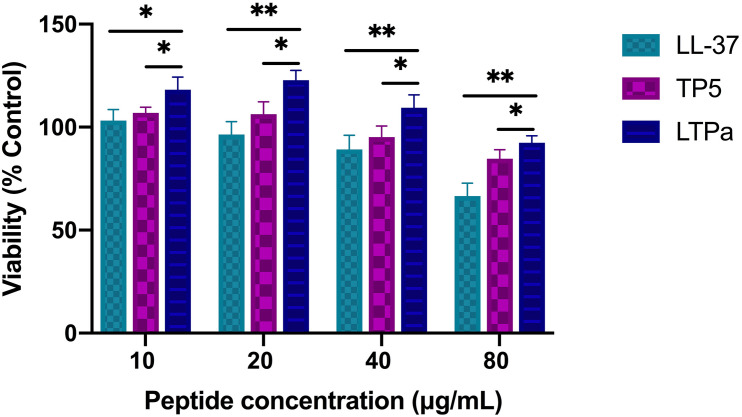
Cell proliferation rates of RAW264.7 cells. RAW264.7 macrophages were treated with various doses of peptides (10, 20, 40, and 80 μg/mL) at 37°C and 5% CO_2_ for 24 h. Cell proliferation rates were measured by microplate at 450 nm using CCK8 assay. The mean values ± SD from 12 independent experiments were used to express the data. ^∗^*p* ≤ 0.05, and ^∗∗^*p* ≤ 0.01.

The cytotoxic activity of LTPa and its parental peptides (LL-37 and TP5) toward RAW264.7 macrophages was evaluated by CCK-8 assay. As shown in Figure 3, LTPa exhibited no significant cytotoxicity even at 80 μg/mL. In addition, the results showed that the cytotoxicity of LTPa was lower than that of its parental peptides (LL-37 and TP5).

### Bodyweight and Immune Organ Indices

**FIGURE 4:**
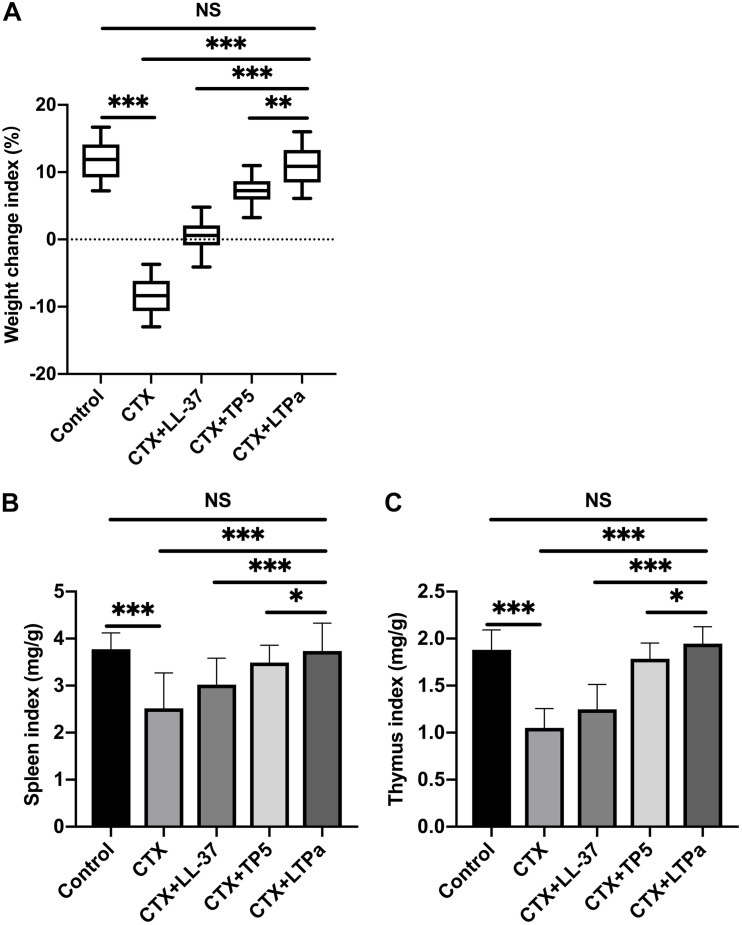
The effects of LTPa on body weight **(A)**, spleen index **(B)**, and thymus index **(C)**. After a 7-day adaptation period, the mice were randomly divided into five groups (*n* = 12 in each group): control group: physiological saline was injected intraperitoneally once daily; cyclophosphamide (CTX) group: CTX (80 mg/kg mouse weight) was injected intraperitoneally once daily for the first 3 days. From days 4 to 10 (7 days), physiological saline was injected intraperitoneally into mice once daily; CTX + peptide (LL-37, TP5, or LTPa) group: CTX (80 mg/kg mouse weight) was injected intraperitoneally once daily for three consecutive days. From days 4 to 10, peptide (10 mg/kg mouse weight) was injected intraperitoneally into mice once daily. The body weight was recorded before and after the experiment. The spleen weights and thymus weights of the mice were examined after 10 days of treatment. The mean values ± SD of 12 biological replicates were used to express the data. NS, *p* > 0.05; ^∗^*p* ≤ 0.05; ^∗∗^*p* ≤ 0.01; and ^∗∗∗^*p* ≤ 0.001.

As expected, there was a significant decrease in the bodyweight of the CTX-treated group compared with the control group (Figure 4A). However, after 7 days of treatment, LL-37, TP5, and LTPa improved bodyweight rapidly. In addition, bodyweight in the LTPa-treated group was significantly higher than that in the parental peptide (LL-37 and TP5)-treated groups (Figure 4A). In addition, the results showed that compared with the control group, the spleen (Figure 4B) and thymus (Figure 4C) indices were significantly decreased in the CTX-treated group. Peptide treatment remarkably improved the spleen (Figure 4B) and thymus (Figure 4C) indices, and the immune organ indices in the LTPa-treated group were significantly higher than those in the LL-37-treated and TP5-treated groups.

### Effect of LTPa on T Lymphocyte Subpopulations

**FIGURE 5:**
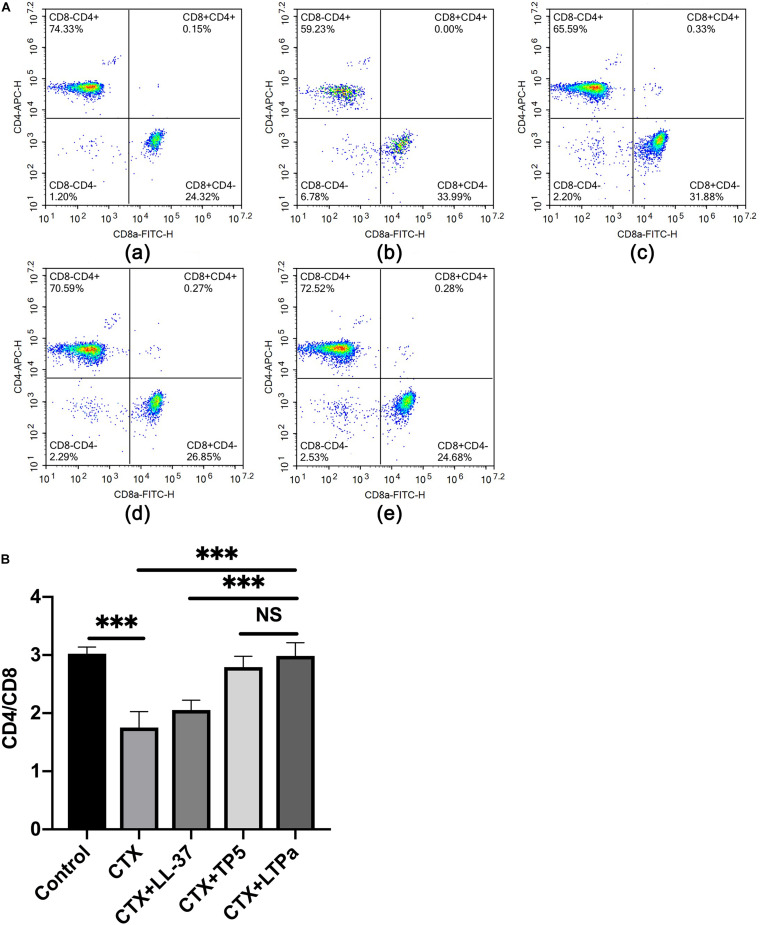
Effects of LTPa on T lymphocyte subpopulations. Erythrocytes were aseptically obtained from the different groups of mouse peripheral blood and lysed with RBC lysis buffer. The cells were combined with PerCP-conjugated anti-mouse CD3 +, APC-conjugated anti-mouse CD4 +, and FITC-conjugated anti-mouse CD8 + for 30 min at 4°C. The T lymphocyte subpopulations were analyzed by flow cytometry. **(A)** The percentage of different T cell subsets was analyzed by flow cytometry. **(A,a)** Control, **(A,b)** cyclophosphamide (CTX), **(A,c)** LL-37 + CTX, **(A,d)** TP5 + CTX, **(A,e)** LTP_a_ + CTX. Bivariate plots are shown as representative, independent assessments that were quantified and plotted as the CD4+:CD8+ ratio in part **(B)**. The mean values ± SD of three biological replicates were used to express the data. NS: *p* > 0.05, and ^∗∗∗^*p* ≤ 0.001.

Compared with the control group, CTX remarkably reduced the proportions of CD4 + and CD8 + T lymphocytes (Figures 5A,B). Treatment with peptides significantly increased the proportions of CD4 + and CD8 + T lymphocytes. In addition, the increasing level in the LTPa-treated group was significantly higher than that in the LL-37-treated group and similar to that in the TP5-treated group.

To evaluate the anti-inflammatory effect of CTP and its parental peptides, CATH2 and TP5, RAW264.7 cells were used as a model. The results showed that LPS caused significant elevation of the pro-inflammatory cytokines TNF-α (Figure 2A), IL-6 (Figure 2B), and IL-1β (Figure 2C) compared with untreated cells. As shown in Figures 2A–C, all the peptides attenuated the TNF-α, IL-1β, and IL-6 secretion levels. Furthermore, compared with its parental peptides, CTP exerted enhanced inhibitory activity against LPS-induced inflammation.

### Effect of LTPa on DC Maturation

**FIGURE 6:**
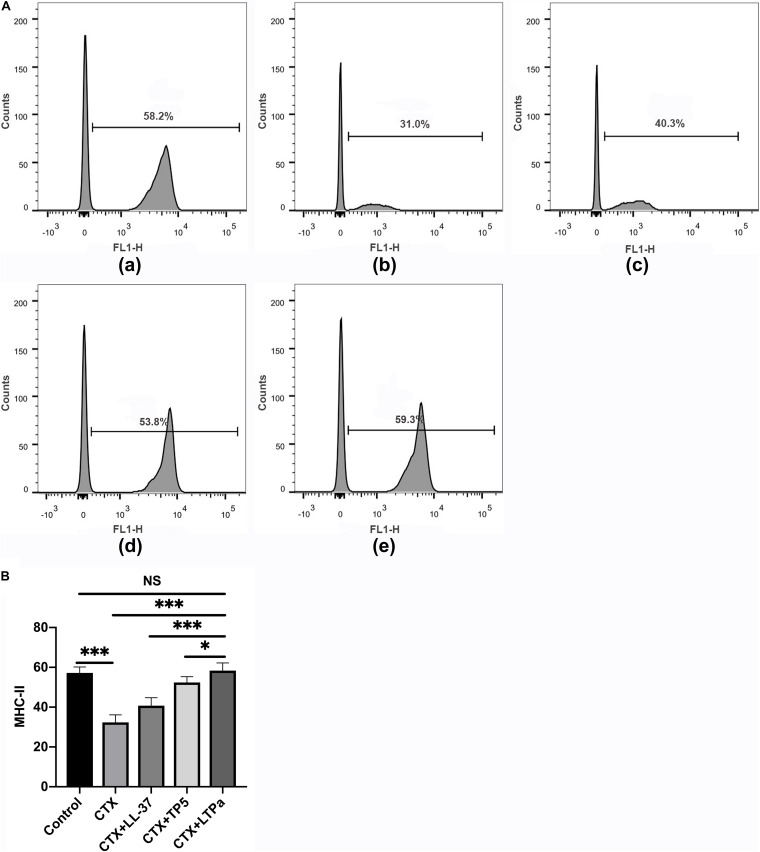
Effects of LTPa on DC maturation. Mouse PBMCs were obtained from the different groups of mice. The cells were combined with FITC-conjugated antibodies specific for MHC class II. The surface expression of MHC-II was determined by flow cytometry. **(A)** The percentage of different T cell subsets was analyzed by flow cytometry. **(A,a)** Control, **(A,b)** cyclophosphamide (CTX), **(A,c)** LL-37 + CTX, **(A,d)** TP5 + CTX, **(A,e)** LTP_a_ + CTX. Bivariate plots are shown as representative, independent assessments that were quantified and plotted as the CD4+:CD8+ ratio in part **(B)**. The mean values ± SD of three biological replicates were used to express the data. NS: *p* > 0.05, ^∗^*p* ≤ 0.05, and ^∗∗∗^*p* ≤ 0.001.

To investigate the effect of LTPa on serum DC maturation, the expression levels of DC phenotype factor (MHC-II) were determined by flow cytometry. The results showed that the expression of MHC-II was significantly decreased in the CTX-treated group but increased by LTPa treatment (Figure 6). In addition, the expression of MHC-II in the LTPa-treated group was significantly higher than that in the LL-37-treated and TP5-treated groups (Figure 6).

### Effects of LTPa on Serum Cytokines and Immunoglobulin

**FIGURE 7:**
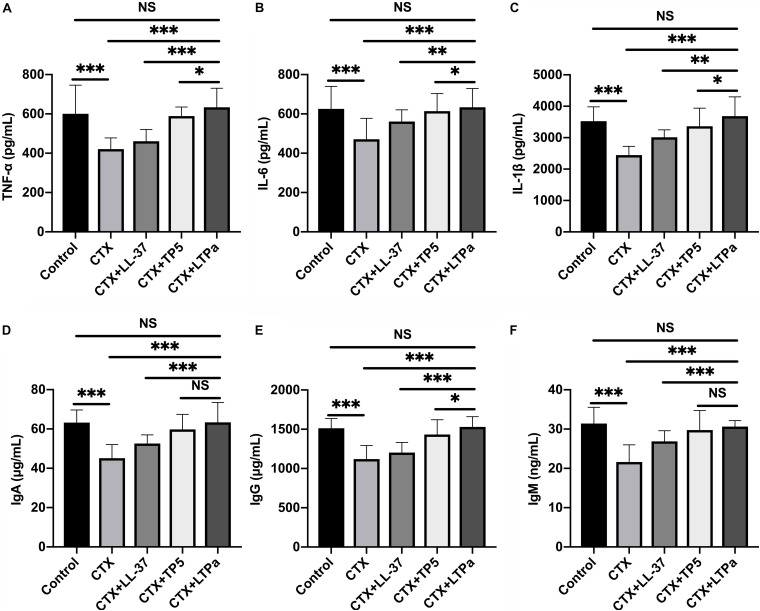
Effects of LTPa on serum cytokines and immunoglobulin. Levels of TNF-α **(A)**, IL-6 **(B)**, IL-1β **(C)**, IgA **(D)**, IgG **(E)**, and IgM **(F)** were detected in serum from different groups of mice through ELISA. The mean values ± SD of three biological replicates were used to express the data. NS, *p* > 0.05, ^∗^*p* ≤ 0.05, ^∗∗^*p* ≤ 0.01, and ^∗∗∗^*p* ≤ 0.001.

To investigate the immunomodulatory activity of LTPa in CTX-treated mice, serum TNF-α, IL-6, and IL-1β levels were evaluated by ELISA. As shown in Figures 7A–C, serum TNF-α (Figure 7A), IL-6 (Figure 7B), and IL-1β (Figure 7C) levels were significantly decreased due to treatment with CTX. However, peptide administration caused a significant increase in the serum TNF-α (Figure 7A), IL-6 (Figure 7B), and IL-1β (Figure 7C) levels compared with the CTX-treated group. In addition, in the LTPa-treated group, TNF-α, IL-6, and IL-1β levels were higher than those in the TP5- or LL-37-treated groups.

The levels of serum IgA (Figure 7D), IgG (Figure 7E), and IgM (Figure 7F) in the CTX-treated group were significantly reduced compared with those in the control group. All of the peptides tested reversed the declines in serum IgA (Figure 7D), IgG (Figure 7E), and IgM (Figure 7F), and LTPa-treated mice had significantly higher total serum IgA, IgG, and IgM levels than mice in the TP5- or LL-37-treated groups.

### LTPa Interacts Directly With TLR2 to Activate the TLR2-NF-κB Signaling Pathway

**FIGURE 8:**
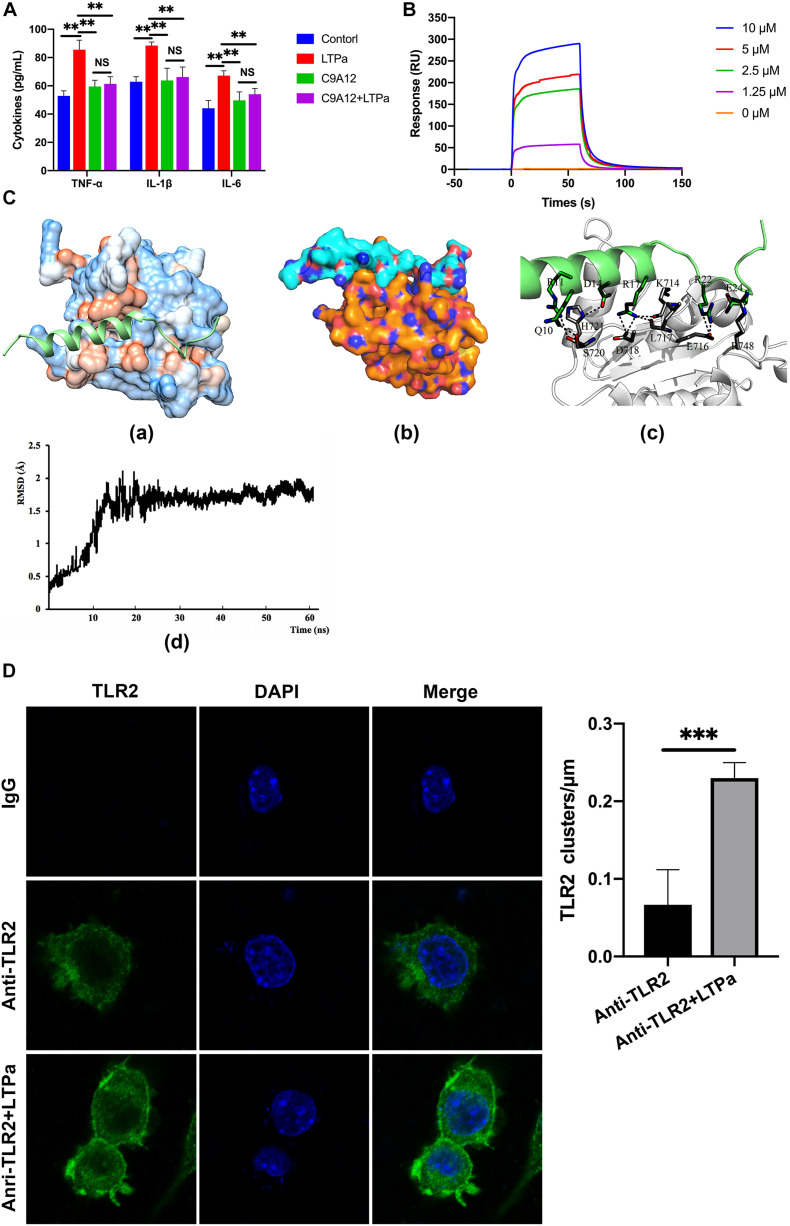
LTPa exhibited its immunomodulatory activity by interacting with TLR2. **(A)** RAW264.7 cells were incubated with PBS or TLR2 mAb (C9A12) for 1 h, followed by treatment with or without 10 μg/mL LTPa for 24 h. Then, the concentrations of TNF-α, IL-6, and IL-1β in the cell culture supernatant were quantified by ELISA. **(B)** TLR2 was immobilized on a sensor chip, and the binding ability of LTPa was analyzed by surface plasmon resonance. **(C)** Docking results of LTPa on TLR2. (C-a) The whole view of TLR2 depicting the LTPa binding pocket; (C-b) electrostatic potential surface around the TLR2-LTPa complex; (C-c) closeup view of LTPa binding to TLR2. The interacting residues between LTPa and TLR2 are shown. The gray ribbons represent TLR2, and green ribbons represent LTPa. (C-d) Time evolution of RMSD during molecular dynamics simulation. **(D)** LTPa enhances cell surface TLR2 cluster formation. RAW264.7 cells were treated with anti-mouse monoclonal antibody TLR2 or isotype control (IgG) in the absence or presence of the indicated LTPa (10 μg/ml). The cells were then washed with PBS and stained with a FITC–conjugated anti-mouse IgG antibody. After washing with PBS and fixing with paraformaldehyde, the cell nuclei were stained with DAPI. The cells were then imaged with the Leica TCS SP5 confocal system. LAS AF software was used to process the images and quantify the fluorescence. In addition, to generate a fluorescence histogram profile, a line was drawn along the cell surface. Fluorescence intensities higher than 40 arbitrary units (isotype control staining) were considered clusters of TLR2 molecules. The mean values ± standard deviation of three independent experiments were used to express the data. NS: *p* > 0.05, ^∗∗^*p* ≤ 0.01, and ^∗∗∗^*p* ≤ 0.001.

A total of 300 snapshots were observed from the last stable 40 ns of the MD simulation for the LTPa-TLR2 complex to understand how LTPa may interact with TLR2. The predicted binding free energy was used to reflect the binding affinity of LTPa. Table 2 represents the predicted binding energy of LTPa to be −798.5 kJ/mol. In addition, the interaction between LTPa and TLR2 primarily involved hydrogen bonds and salt-bridges (Figure 8C and Table 3).

**TABLE 2:** Key interaction parameters between LTPa and TLR2.

Interaction Pair	Number of Hydrogen bonds	Number of Salt-bridges	Interaction Surface (Å^2^)	Binding free energy (kj/mol)
TLR2…LTPa	11	7	293	−798.5

**TABLE 3:** Distance and salt-bridges of binding residues between LTPa and TLR2.

Interaction Pair TLR2…LTPa	Distance (Å)	Number of salt-bridges
R748…E24	2.50	2
E716…R22	2.61	2
K714…R17	2.98	0
L717…R17	3.00	0
D718…R17	3.12	2
H721…D14	3.30	1
S720…R11	2.89	0
S720…Q10	2.97	0

To explore the mechanism underlying the capacity of LTPa to interact with TLR2, we first examined cell surface TLR2 cluster formation because TLR clustering is believed to be critical to signaling (Pfeiffer et al., 2001; Triantafilou et al., 2002). In the absence of LTPa, TLR2 showed an even distribution on the cell surface with very few clusters per micrometer (Figure 8D). However, LTPa significantly increased the number of major TLR2 clusters per micrometer on the cell surface (Figure 8D).

**FIGURE 9:**
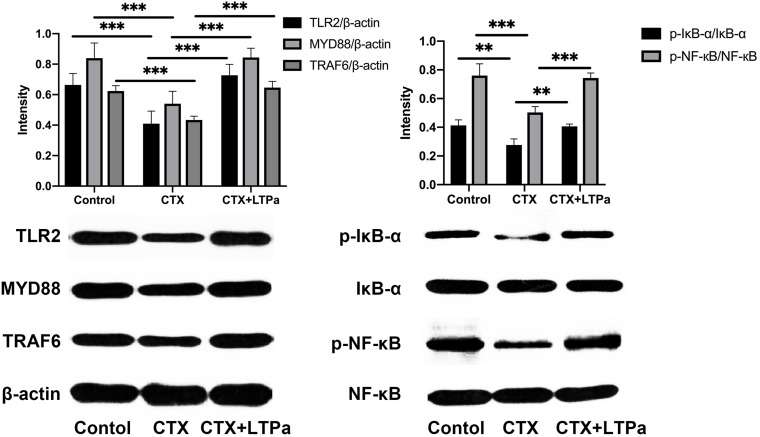
Effect of LTPa on the TLR2-NF-κB signaling pathways. Phosphorylated and total protein levels of TLR4, MyD88, TRAF6, β-actin, IκB-α and NF-κB from serum were measured by western blot analysis. The mean values ± standard deviation of at three independent experiments has been used to express the data. ^∗∗^*p* ≤ 0.01, and ^∗∗∗^*p* ≤ 0.001.

Next, the TLR2-NF-κB signaling pathway was investigated to determine the mechanism underlying the immunomodulatory activity of LTPa. CTX significantly decreased the expression levels of TLR2, MyD88, and TRAF6 and the phosphorylation of IKK-β, IκB-α, and NF-κB, while cells that were treated with LTPa exhibited dampened levels of TLR2, MyD88, and TRAF6 and phosphorylation of IKK-β, IκB-α, and NF-κB (Figure 9). These results suggest that LTPa exerts its immunomodulatory activity by activating the TLR2-NF-κB pathway.

## Discussion

As a state of temporary or permanent immune dysfunction, immunosuppression can make an organism more susceptible to infection, organ injury, and cancer (Goldszmid et al., 2014). Development of new immunomodulatory agents to prevent and treat immunosuppressive diseases has taken a long time. Meanwhile, in recent years, immunomodulatory peptides have been reported to have profound effects on the immune system, thereby being regarded as potent immunomodulatory candidates with a wide range of applications (Nijnik and Hancock, 2009; Chen et al., 2010; Li et al., 2012; Romani et al., 2017). Among them, TP5 and LL-37 have displayed enormous potential in the treatment of various immunosuppressive diseases (Nijnik and Hancock, 2009; Chen et al., 2010). However, their development has been weakened by several concerns, such as potential cytotoxicity (Anders et al., 2018), weak physiological stability and poor immunomodulatory activity (Gonser et al., 2004; Hu et al., 2016). To overcome all these challenges, hybridization has been proposed (Liu Y.F. et al., 2013; Ma et al., 2015; Zhang et al., 2019a,b). Therefore, six hybrid peptides were designed by combining the full-length TP5 (19, 20) with a characteristic fragment of LL-37 that included LL-37 (13–36) (Mookherjee et al., 2006; Molhoek et al., 2009), LL-37 (17–29) (Li et al., 2006; Tan et al., 2017), and LL-37 (13–31) (Wei et al., 2018).

TLR2 plays prominent roles in host defense and in regulating immune responses (Komai-Koma et al., 2004). TLR2 is an important signal transduction molecule that is expressed on DCs, macrophages and activated CD4 + T cells (Komai-Koma et al., 2004). TLR2 activates the secretion of pro-inflammatory cytokines such as TNF-α, IL-6, and IL-1β from monocytes, macrophages and dendritic cells, thereby triggering the activation of the immune response (Oliveira-Nascimento et al., 2012). Therefore, targeting TLR2 is an important therapeutic strategy against immunosuppressive diseases. Initially, the binding modes of the hybrid peptides were simply and effectively screened by molecular docking. Among the six newly designed peptides, LTP_*a*_ had the most favorable docking scores for TLR2. In addition, the immunomodulatory activity of the hybrid peptides was also confirmed by *in vitro* experiments. The results showed that LTPa significantly increased the secretion of TNF-α, IL-6, and IL-1β in comparison with the other peptides tested. Collectively, these data suggested that LTPa, the most active peptide, should be selected for further immunomodulatory experiments.

The hybrid peptide was designed to have potent immunoregulatory activity but minimal cytotoxicity. Thus, the cytotoxic activity of the hybrid peptides were evaluated by the CCK-8 assay. The results revealed that LTP_a_ had lower cytotoxicity than its parental peptides.

CTX, as a typical immunosuppressant, that can interfere with the differentiation and proliferation of T and B cells. Furthermore CTX kills immune cells, and decrease cellular and humoral immune responses (Fan et al., 2013; Ren et al., 2014; Gong et al., 2015). Thus, the CTX-immunosuppressed murine model was used to evaluate the immunoregulatory effects of LTP_a_. The spleen and thymus play prominent roles in non-specific immunity, so their indices are crucial features of immune function (Mei et al., 2013). As expected, there was a significant decrease in the body weight and spleen and thymus index values of the CTX-treated group compared with the control group. However, peptide treatment remarkably improved the body weight and spleen and thymus indices, and these indices in the LTPa-treated group were significantly higher than those in the LL-37-treated and TP5-treated groups.

As primary helper and effector cells in the adaptive immune response, T lymphocytes are important for the regulation of the immune response (Yun et al., 2018). When the immune system is suppressed, the organism is more susceptible to infection due to the decrease in the CD4 + : CD8 + ratio (Fan et al., 2018). Consistent with a previous study, CTX remarkably reduced the proportions of CD4 + and CD8 + T lymphocytes. Treatments with peptides significantly increased the proportions of CD4 + and CD8 + T lymphocytes, and the increasing level in the LTPa-treated group was significantly higher than that in the LL-37-treated group. These results suggested that LTP_a_ improved immune function by regulating T lymphocyte subsets.

DCs are the most potent antigen-presenting cells that can regulate the activation and differentiation of T cells (Nace et al., 2012). It has been reported that mature DCs upregulate MHC-II, display costimulatory molecules and produce cytokines, thereby playing an important role in immune induction and regulation (Lambrecht and Hammad, 2010; Liu et al., 2015). Thus, DCs are potential targets for therapeutic intervention in immunosuppressive diseases (Liu et al., 2015). In this study, we determined the expression levels of MHC-II to investigate the effect of LTPa on serum DC maturation. The results showed that the MHC-II level was significantly decreased in the CTX-treated group but increased by LTPa treatment. In addition, the expression of MHC-II in the LTPa-treated group was significantly higher than that in the LL-37-treated and TP5-treated groups, indicating that LTPa could enhance DC maturation in CTX-induced immunosuppressed mice.

Cytokines are the key players in cell-cell communication in the immune system (Liu et al., 2016). Cytokines, such as TNF-α, IL-6, and IL-1β, are involved in immune regulation by coordinating hematopoietic, inflammatory, and lymphoid cells (Mei et al., 2013). The present study showed that treatment with LTPa efficiently reversed the declines in TNF-α, IL-6, and IL-1β levels caused by CTX. In addition, we found that the immunomodulatory activity of LTPa was higher than that of its parental peptides.

Immunoglobulins, such as IgA, IgG, and IgM, are involved in complement activation, opsonization, and toxin neutralization (Yu et al., 2014). Levels of serum IgA, IgM, and IgG in the CTX-treated group were significantly reduced compared with those in the control group. However, pretreatment with LTP_a_ significantly reversed the reduction. Furthermore, it is worth noting that LTPa-treated mice had significantly higher total serum IgA, IgG, and IgM levels than mice in the TP5- or LL-37-treated groups.

Collectively, these results indicate that the newly designed hybrid peptide LTPa has more potent immunoregulatory activity than its parental peptides and minimal cytotoxicity. To identify the underlying immunomodulatory mechanisms, a comprehensive and detailed analysis was conducted.

Firstly, to investigate whether the LTPa exhibited immunomodulatory properties by binding to the TLR2 as proposed, binding assays were performed by ELISA. The results showed that treatment with LTPa significantly caused increase levels of TNF-α, IL-6, and IL-1β, but pre-treatment with TLR2 mAb significantly inhibited the TNF-α, IL-6, and IL-1β production induced by LTPa, indicating that LTPa exhibits its immunomodulatory effects through the TLR2 receptor. Furthermore, the binding ability of LTPa was analyzed by SPR assay, and the results confirmed that LTPa could effectively bind to the TLR2. In addition, to identify the binding sites of LTPa, we mapped the docking process of the LTPa-TLR2 complex by MD simulation. The results showed that LTPa can establish sustainable interaction with TLR2 by the hydrophobic interactions, which is crucial for initiating immune responses *via* TLRs (Seong and Matzinger, 2004). In addition, the TLR2-LTPa complex is completely stable during the simulation time. Overall, these results suggested that LTPa exhibited its immunomodulatory effects depending upon binding to TLR2.

TLR2 was found to form clusters and to engage its ligand on the cell membrane where signaling was initiated (Pfeiffer et al., 2001; Triantafilou et al., 2002; Peddireddy et al., 2016). Thus, in the present study, we examined cell surface TLR2 cluster formation by confocal laser-scanning microscopy. The results showed that LTPa significantly increased the number of major TLR2 clusters per micrometer on the cell surface, which indicated that LTPa enhanced immune function by forming TLR2 clusters.

The NF-κB signaling pathway plays a crucial role in host defenses through regulation of inflammatory gene expression (Hayden and Ghosh, 2004). MyD88 is utilized by TLR2 and recruits TRAF6, and then activates NF-κB signaling (Kawai and Akira, 2010). This work showed that the expression of the major proteins in the NF-κB pathway was assessed to elucidate the immunomodulatory mechanism of LTPa. LTP_a_ significantly increased the expression of TLR2, MyD88, and TRAF6 and the phosphorylation of IκB-α and NF-κB, indicating that LTP_a_ could successfully activate the TLR2-NF-κB pathway.

## Conclusion

**FIGURE 10:**
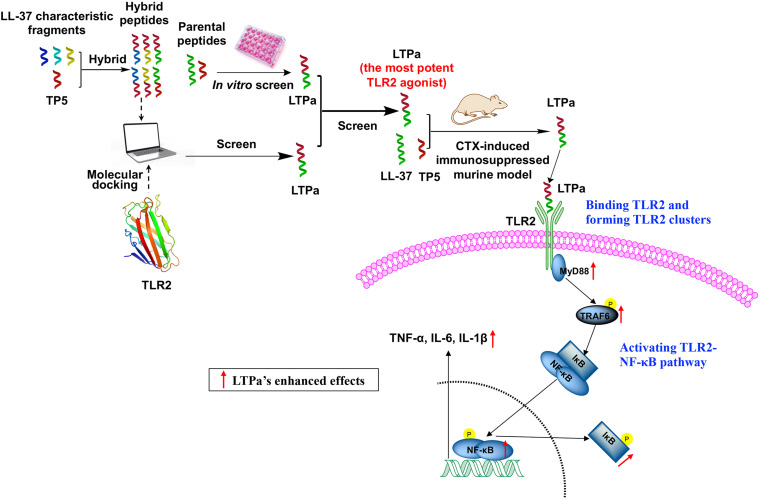
Schematic illustration of the design and development of the novel hybrid peptide LTPa.

The successful design of LTP_a_ may provide a feasible approach to design novel active peptide agents with desired immunomodulatory activity and minimal cytotoxicity (Figure 10). Initially, six hybrid peptides (LTA_a_, LTA_b_, LTA_c,_ TPLa, TPLb, and TPLc) were designed by combining the full-length TP5 with a characteristic fragment of LL-37. LTPa, the most active peptide, was simply and effectively screened by molecular docking and *in vitro* experiments. Furthermore, the immunomodulatory effects of LTPa were confirmed by a CTX-immunosuppressed murine model, which showed that LTPa successfully inhibited immunosuppression, increased immune organ indices, enhanced DC maturation, regulated T lymphocyte subsets, and increased cytokines and Ig contents. The present study revealed that the immunomodulatory effects of LTPa are associated with binding to the TLR2, forming TLR2 clusters, and activating the NF-κB signaling pathway.

## Data Availability Statement

The original contributions presented in the study are included in the article/Supplementary Material, further inquiries can be directed to the corresponding author/s.

## Ethics Statement

The animal study was reviewed and approved by Institutional Animal Care and Use Committee of China Agricultural University.

## Author Contributions

LZ, XW, RZ, PM, and DS conceived the project and designed the experiments. LZ, XW, DS, BA, QC, and YT conducted experiments. LZ wrote the manuscript and analyzed data. All authors read and commented on the manuscript.

## Conflict of Interest

The authors declare that the research was conducted in the absence of any commercial or financial relationships that could be construed as a potential conflict of interest.

## Footnotes

1 http://www.expasy.org/tools/protparam.html

2 http://zhanglab.ccmb.med.umich.edu/I-TASSER/

3 http://flexpepdock.furmanlab.cs.huji.ac.il/

## Abbreviations

TLRs: Toll-like receptors
DCs: Dendritic cells
TNF- α: tumor necrosis factor-alpha
IL-6: interleukin-6
TP5: thymopentin
CTX: cyclophosphamide
LTPa: LL-37-TP5a
LTPb: LL-37-TP5b
LTPc: LL-37-TP5c
TPLa: TP5-LL-37a
TPLb: TP5-LL-37b
TPLc: TP5-LL-37c
HPLC: high-performance liquid chromatography
MS: mass spectrometry
DMEM: Dulbecco’s modified Eagle’s medium
FBS: fetal bovine serum
CCK-8: Cell Counting Kit-8
PerCP: Peridinin-Chlorophyll-Protein Complex
APC: Allophycocyanin
FITC: fluorescein isothiocyanate
PBMCs: eripheral blood mononuclear cells
MHC-II: MHC class II
MD: molecular dynamics
NPT: constant number pressure and temperature
NVT: constant number volume and temperature
MM-PBSA: Poisson-Boltzmann accessible surface area
NF- κ b: nuclear factor-kappa B
CE TAL: Chromogenic End-point Tachypleus Amebocyte Lysate
MD: molecular dynamics
SPR: Surface plasmon resonance
*K*a: kinetic rate constant
*K*d: the dissociation rate constant
*K*D: equilibrium dissociation constant.
