# Supplementary figures and images for: Design and Immunological Evaluation of a Hybrid Peptide as a Potent TLR2 Agonist by Structure-Based Virtual Screening
Source: Front Cell Dev Biol. 2021 Feb 11;9:620370. doi: 10.3389/fcell.2021.620370 (PMC7905067; doi:10.3389/fcell.2021.620370)

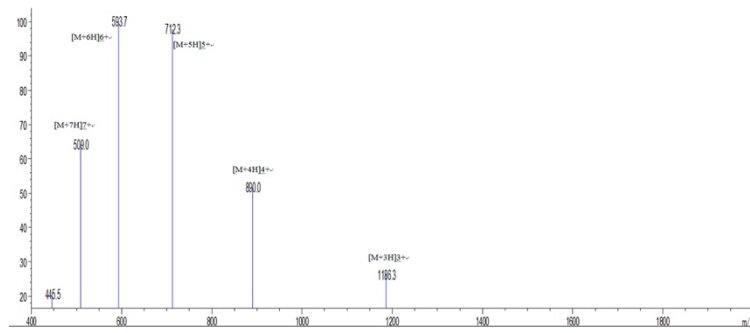

**Figure S1.** Mass spectrometry analysis of purity peptides.

Supplement: Supplementary file 1 [file Image_1.pdf]
